# “No More Buzz”: A Qualitative Study of the Current Response to HIV in the Anglican Church in the Western Cape, South Africa

**DOI:** 10.1007/s10943-017-0397-x

**Published:** 2017-04-19

**Authors:** Simon Hallonsten

**Affiliations:** 1 Department of Theology, Uppsala University, Uppsala, Sweden; 2 56 Hobson Street, Boston, MA 02135 USA

**Keywords:** HIV, South Africa, Anglican Church, Stigma

## Abstract

Using a triangulation design combining participant observation, survey results, and interviews, this paper studies the current form of the response to HIV in the local Anglican Church in the Western Cape, South Africa. The results show that people are generally aware of HIV. The “buzz” around HIV has, however, subsided. The local church does not directly engage HIV anymore, and HIV is more mentioned than talked about. HIV stigma continues to pose a challenge. To work towards the prevention of HIV, the local church needs to put HIV back on the agenda and continue to speak about the virus.

## Introduction

At the global level, the response to HIV shows positive trends: fewer people are being infected, fewer people die of HIV-related causes, and more people than ever have access to treatment (UNAIDS 2015). Still, the virus keeps spreading. In South Africa, 380 000 new infections were reported in 2015 (UNAIDS, n.d.). Thus, there is a persistent demand for HIV prevention and a continued need to understand the development of the response to HIV. Part of the overall response is the important role faith communities play in addressing the HIV epidemic (Haddad 2011).

One way in which faith communities influence the response to HIV is through their impact on HIV stigma (Keikelame et al. 2010). Stigma is best defined as an attitude towards someone on the basis of perceived differences and the individual’s deviation from social norms (Liamputtong 2013). These attitudes are contextual, based on a complex set of social, cultural, religious, racial, gender, sexual, and historical aspects (Olivier et al. 2006). Stigma manifests itself in actions, such as verbal abuse, gossip, and the distancing from people living with HIV. Campbell et al. (2006) argue that HIV stigma results from fear of infection coupled with poor information about transmission, fear of poverty, and the association of sexuality with immorality and sin. Liamputtong (2013) sees HIV and AIDS stigma as rooted in the association of HIV and AIDS with immorality, promiscuity, perversion, contagiousness, and death, coupled with a lack of appropriate knowledge about the virus. These associations can lead to the perception of HIV as a punishment from God.

Attitudes towards HIV are based on people’s conceptions of HIV, which is formed and reformed through *HIV talk*—the speaking of HIV and HIV-related things. Understanding HIV from within a scientific, a religious, or a cultural framework radically alters the behavioural responses. Depending on whether a person understands the cause of HIV as a virus transmission, a punishment from God, or bewitchment, different prevention strategies are chosen and different treatment options considered, such as antiretroviral therapy, prayer and repentance, or healing and the fighting of evil spirits (Benn 2002).

Stigma also affects HIV prevention. Stigmatised persons often employ coping strategies such as secrecy, denial, deception, and social withdrawal in order to avoid rejection (Greeff 2013). Stigma is furthermore a barrier to voluntary testing and counselling (VTC) (Mall et al. 2013). With the importance of knowing one’s status in the epidemic, obstacles to testing increase the risk of transmission as people are less likely to make the necessary prevention efforts and seek treatment. Stigma also impacts on the willingness of people living with HIV to access health services and adhere to treatment (Peretti-Watel et al. 2006; Keikelame et al. 2010). Treatment in turn affects prevention in at least two ways. First, appropriate antiretroviral drugs can reduce the viral load of people living with HIV to such a degree that the probability of passing the virus to a sexual partner becomes minimal (Messer 2011). Second, treatment is correlated with the normalisation of HIV within sociocultural contexts (Roura et al. 2009). Normalisation reduces the stigma attached to HIV by altering the perception of HIV as a deviation from the norm and thus enables people to access health services without fear of rejection. In the absence of treatment, however, people living with HIV are susceptible to opportunistic diseases, which negatively impact their health and well-being. As health deteriorates and the infection and its consequences become visible, fear of contagion and a discourse of blame set in, which further add to HIV stigma (Benn 2002; World Council of Churches 2006; Ragnarsson et al. 2009).

As stigma is rooted in conceptions of the normal, stigma can be broken by information, openness, and understanding, which lead to the normalisation of HIV. Speaking openly about HIV and AIDS reduces stigma, increases the uptake of VTC, increases the engagement in treatment, and makes it possible for people to assess their own exposure more accurately (Mall et al. 2013; Roura et al. 2009; Keikelame et al. 2010). Public self-disclosure is another mechanism to bring about normalisation and is found to be negatively correlated with HIV stigma (Smith et al. 2008).

In South Africa, one of the social contexts in which conceptions and understandings of HIV are formed is the local church, which is often seen as an important place for information (Schmid 2006). Churches play a role in the perception and conceptualisation of HIV by engaging in *HIV talk*. Keikelame et al. (2010) conducted key informant interviews in Cape Town, Durban, Pretoria, and Johannesburg in 2010 with leaders in the Anglican, Moravian, Methodist, and Presbyterian churches, and traditional indigenous and Muslim leaders. The authors find that stigma continues to be a problem and that some religious leaders and organisations are propagating stigma, equating HIV with sin and punishment from God. Keikelame et al. also demonstrate that there is a difficulty among religious leaders to address questions of sex and sexuality, even as faith communities have undertaken important steps to fight stigma. Visser and Sipsma (2013) show that HIV and AIDS is perceived in negative terms and connected to stigma by both HIV-infected women and community members in Tshwane, South Africa, three decades into the epidemic. Barney and Buckingham (2012) conducted a qualitative study of the interplay between spirituality and HIV and AIDS in a township in Johannesburg. They establish a complex picture; the understanding of God and the church, ancestral spirits, and bewitchment all contribute to the understanding of and coping with HIV and AIDS. They can be a positive resource in people’s lives, but they can also be condemning, leading to stigmatisation and isolation. In agreement with Keikelame et al., Barney and Buckingham see that there is a tendency to avoid the topic of HIV in the churches. Eriksson (2011) studied HIV prevention methods and their effect on youth sexuality in the Roman Catholic Church, the Lutheran Church, and the Assemblies of God in KwaZulu-Natal, South Africa, and shows that religious leaders struggle with breaking the silence around HIV. HIV prevention messages are often ambivalent as sexuality is omitted in the churches. Church-attending young people, while seeing the church as an important institution, often understand church teachings as focussed mainly on abstinence and report that even though churches do engage in education, basic questions of HIV transmission remain unclear to them.

One of the churches that early declared its willingness to address HIV stigma is the Anglican Church of Southern Africa (ACSA). The strong dedication to break the silence around HIV and its commitment to respond to HIV and AIDS make ACSA an interesting case for studying the long-term contribution of churches in the response to HIV and AIDS. The main focus in this paper is on the Anglican parish of St. Clare of Assisi in Ocean View, Cape Town. The current form of the parish’s response to and perceptions of HIV are examined through a combination of participant observation, interviews, and survey responses. The emerging picture is then expanded by survey results from two other parishes in the greater Cape Town area and clergy in the dioceses of Cape Town and False Bay. The extended case study approach aims at both providing a detailed study of the local engagement and at establishing more general insights into the current state of the response to the HIV epidemic in the Anglican Church of Southern Africa in the Western Cape. The paper thus seeks to answer the question how the local response to HIV has developed in the last decade.

## Background

In the first years of the twenty-first century, the Anglican Church of Southern Africa (ACSA) issued a number of statements committing itself to “breaking the silence in order to end all new infections” and to “ending stigma and judgement” (Anglican Communion across Africa 2002). ACSA made HIV and AIDS a priority area at the 2001 synod meeting, and Fikelela AIDS Project was established as a specialised HIV office in the Cape Town diocese the same year. Fikelela aims at mobilising the Anglican Church through the forming of local HIV and AIDS Task Groups. HIV and AIDS groups were to put HIV and AIDS onto the local church agenda and encourage participation by church members. Funding for local initiatives was made available through the establishment of the Anglican AIDS and Healthcare Trust (AAHT) in 2003, which was initially supported by a grant from the British Government’s Department for International Development (Mark et al. 2012).

In April 2005, 92 congregations had established HIV Task Groups out of 136 parishes that were part of the diocese of Cape Town (Kareithi et al. 2005). The same year Fikelela commissioned a study on the sexual behaviour of Anglican youth (aged 12–19 years) in the Western Cape. The results show that Anglican youth are sexually active and that condom use is generally low (Mash et al. 2006). In response to the study findings, Fikelela started the *Agents of Change* peer educators programme to train young people to become peer educators and role models (Fikelela AIDS Project 2015). The programme raises the age of sexual debut for young people and increases the use of condoms (Mash and Mash 2012).

While additional funding was made available between 2007 and 2012 through a grant from USAID, international funding subsided as South Africa started to increase domestic HIV spending. In 2014, South Africa mostly funded its own response to HIV and AIDS (USAID 2012; AVERT 2017). However, faith-based organisations, such as AAHT, have received comparably little domestic government funding, to the point that the AAHT was discontinued and responsibility for the coordination of the Anglican response to HIV integrated into Hope Africa, a diocesan organ to address the developmental needs in the diocese (Mark et al. 2012; Olivier and Smith 2016). The larger initiatives, such as Fikelela, who had diversified their donor base, were able to continue their work. However, many of the smaller, parish-based initiatives were discontinued when funding subsided.

## Method

Adopting a qualitative approach, the study employs a single time triangulation design in both methods and sources, drawing on participant observation, semi-structured interviews, and survey answers from three different geographic areas (Creswell 2007). The material was collected between 18 March and 17 May, 2016, in the Western Cape, South Africa. Data were gathered in three local congregations of the Anglican Church of Southern Africa (ACSA): Ocean View: St. Clare of Assisi, Lentegeur: Christ the Saviour, and Constantia: Christ Church. An online survey was sent to ACSA clergy in the Cape Town and False Bay dioceses.

### Participant Observation

Participant observation was carried out in the parish of Ocean View: St. Clare of Assisi. The degree of participation was moderate, in which participation is almost complete in activities, but not in culture (Kawulich 2005). The observations consist of eight services held in the congregation on Sunday mornings (8.00 o’clock), Wednesday morning (9.30 o’clock), and Wednesday evening (19.00 o’clock). One of the Wednesday morning services was a healing service. Healing services are held on the first Wednesday every month and include intercession on behalf of the sick and the anointing of afflicted body parts with oil.

### Surveys

A short survey with ten items was distributed in paper form in the three Anglican congregations and sent electronically to 216 Anglican clergy in the dioceses of Cape Town and False Bay. To align as closely as possible with the interview method, the questionnaire contained seven open free-text questions. In total 73 respondents completed the survey. The majority of completed surveys came from the parish of Ocean View (32). The sample sizes from the congregation in Constantia, the congregation in Lentegeur, and the online survey were roughly similar to 12, 15, and 14 returned surveys, respectively. The total sample comprises clergy (15), lay leaders (13), such as lay ministers, parish councillors, wardens, confirmation instructors, and Sunday school teachers, and parishioners (24). Remaining respondents did not report their role in the congregation. The female/male ratio was 47 to 24 which could be explained by generally more women attending church. Age was fairly evenly distributed ranging from 15 to 94 years old. Splitting ages into 10-year age groups, each group up to 80 years was represented by at least three responses. The average age was 50 years. All in all, the survey material is spread over a number of dimensions and can be believed to represent a variety of opinions and experiences. Especially from the perspective of quantitative methods, however, sampling is an issue. As the survey was self-administered, it cannot be assumed that it is a random sample. The material is therefore added to the data gathered through qualitative interviews and used for qualitative analysis.

### Interviews

Lastly, a series of face-to-face semi-structured interviews was conducted to obtain a deeper insight into the understanding of and attitudes towards HIV. A stratified sampling strategy was employed based on age and gender. Interviews were held with clergy, lay leaders, and parishioners from the parishes of Ocean View: St. Clare of Assisi, Lentegeur: Christ the Saviour, and Constantia: Christ Church. Interviews were on average 54 min long. The interviews were held at the local Anglican Church (4), people’s private homes (5), or office (1). One interview was held as a group interview with three participants. Three of the interviews were done in connection with the survey and aimed to elaborate on the survey answers. One interview was conducted with a key informant from Fikelela AIDS project, a diocesan organisation of the diocese of Cape Town specialising in questions of HIV and AIDS. The interview participants aged between 33 years and 63 years. In total seven women were included and five men. Most participants were from the parish of St. Clare of Assisi. Relevant parts of the interviews were transcribed, and the interview material first coded and subsequently categorised to arrive at common themes and conceptions. In a second step, the same was done with the survey material. The categories were then compared and combined. To determine the sufficient size of the interview sample, theoretical saturation was used, which occurs if interviews add nothing essentially new to the issue in question (Davidsson Bremborg 2011). Theoretical saturation was assumed to have been reached when no new codes emerged during coding. This happened after ten interviews.

### Ethical Considerations

An application for the field study was accepted by the Faculty of Theology at Uppsala University as part of the Swedish International Development Cooperation Agency’s (SIDA) Minor Field Study programme. Consent for the participant observation, interviews, and paper surveys was first obtained from the rector of each parish. Informed consent was then obtained from all individual participants included in the study. Interview and survey participants were informed about the objectives of the study and the use and confidentiality of their data. It was also stressed that participation is voluntary and can be withdrawn in part or in full at any time.

## Results

### HIV Awareness

When asked about the main challenges in the lives of members in their congregation, very few people mention HIV. This points to the fact that few people are immediately concerned with HIV. This finding is in line with the fact that there is no one who is publicly known to be HIV+ in any of the three congregations. Perceptions of the seriousness of HIV vary markedly between respondents. Looking at the whole sample, the survey respondents mildly disagreed with the statement “I feel that HIV is an issue in my congregation”. Respondents generally agreed with the statement “I feel that HIV is an issue in my community”, however. Respondents strongly agreed with the statement “I feel that HIV is an issue in South Africa”. This indicates that HIV is generally accepted as a concern. However, HIV is seen as a concern for others, rather than for one’s own congregation.

Another indicator of HIV awareness is the number of people who reported having had an HIV test. In total, 76% of respondents said they had been tested at least once. The percentage in Ocean View and amongst clergy is close to the total sample, 75% and 73%, respectively, while it is somewhat lower in Constantia (64%) and much higher in Lentegeur (93%). Even though the reasons for getting an HIV test are many, from routine check-ups in antenatal care to compulsory HIV tests in certain lines of work, the large extent to which people have had HIV tests shows that many people have come into close contact with HIV issues. This is confirmed in the interview material in which respondents stress the anxiety that an HIV test entails as one is necessarily forced to consider whether one could have contracted the virus.

In Ocean View, 19 out of the 32 survey respondents (59%) reported that HIV is talked about in the congregation. If one assumes that *HIV talk* can only address questions of HIV if it is noted and thus adds the four participants who answered that they do not know whether HIV is spoken of to the nine voices saying that HIV was not addressed, it appears that roughly half of the people see the church to engage in HIV talk. This finding is confirmed in the other three contexts.

The divergent assessments are rooted in different ideas of what constitutes HIV talk. From the interview material, it becomes clear that HIV is mostly addressed around and on World AIDS day on December 1. HIV is also mentioned in services and particularly in the sermon at times. When HIV is talked about, it is most often to raise awareness and to promote acceptance of and compassion towards people living with HIV. However, survey respondents in Ocean View and Constantia also reported that HIV was at times talked about as a concern chiefly for other people.

During the period of participant observation, HIV was mentioned twice in St. Clare. The first time, HIV was mentioned in connection with the introduction of the researcher and the research project. On that occasion HIV + individuals were offered confidential counselling. The other time was in intercession on Mother’s Day, 9 May 2016, when prayer included all mothers infected with HIV.

HIV is, however, rather mentioned than talked about. Survey participants see the unwillingness to seriously engage HIV to be rooted in the sensitive nature of the topic. People are afraid to address the issue, because of stigma, taboo, and ignorance. There is also the feeling that HIV is not relevant as it affects others. One of the apparent wants in the local Anglican Church is that HIV is not publicly self-disclosed. There is agreement among interviewees from Ocean View, Lentegeur, and Constantia that people do not feel comfortable disclosing their status publicly in the local church. The absence of self-disclosures adds to the feeling that HIV is an issue that chiefly pertains to others.

The situation regarding practical HIV work is similar to that of *HIV talk*. The HIV Task Team in Ocean View was formed 2008 with the purpose to provide counselling, support, and care for people and families infected with and affected by HIV. The team also provided food support for people living with HIV. However, no one from the congregation came to disclose their status or to receive counselling, either because there was no need or due to an unwillingness to disclose one’s status. The team subsequently decided to broaden the scope of its commitment in social issue and got involved in three general foster homes. In 2014, it was decided to rename the group to Task Team, thus dropping the HIV.

The experience from Ocean View is mirrored in both Lentegeur and Constantia. Also Christ the Saviour had an HIV Task Team which was eventually discontinued as members became involved in other projects, even though the Women’s Ministry continues to engage in food support for people living with HIV. The same happened to the HIV projects at Christ Church. The fact that of the 92 HIV Task Groups that were active in 2005 only 37 continued to be active in 2015 shows that this is a more general phenomenon (Kareithi et al. 2005; Fikelela AIDS Project 2015).

### HIV Perceptions

While HIV awareness is important in breaking the silence around HIV, it is equally important *what* is said and *how* HIV is understood. In the local Anglican Church, HIV is clearly associated with certain groups, lifestyles, and contexts. These groupings generally describe groups of people who are seen to either have HIV or to be especially at risk of contracting the virus.

During interviews people linked HIV most often to people who sell sex and to promiscuity. The sexual nature of HIV transmission is thus emphasised. Other groups seen as likely to be exposed to HIV are drug users, often because drug use is seen to lead to unprotected and imprudent sex. Interviewees also make a connection between people who sell sex and drug users. Prostitution is understood to be related to poverty, which together with homosexuality, lack of education, and unemployment leads to people being exposed to HIV. Importantly, some interviewees report that people see HIV to only happen to others; the poor, the unemployed, and the badly educated, who sell sex, use drugs, and are generally promiscuous. Other interviewees perceive everybody as being exposed to HIV and understand the virus to affect all people equally. A third assessment relates HIV to the innocent victims of rape, abuse, or unfaithful spouses. Also children are seen to be the innocent victims of HIV.

The survey results confirm the interview findings. In the parish of St. Clare of Assisi, people exposed to HIV are most often perceived to be drug users, those engaging in unprotected sex, and those being unaware of the virus. The results from the parish of Christ the Saviour and Christ Church are similar to respondents focussing on people engaging in unprotected sex, drug use, and promiscuity in Christ the Saviour and unprotected sex and unfaithful partners in Constantia. The clergy deviate from this pattern to some degree with the three groups being seen as most exposed to HIV being young women, the poor, and youth.

Interview participants are well aware of HIV stigma, and many see it as an immense challenge in the HIV response. Stigma is identified as being rooted in a lack of education and knowledge, and ultimately in fear generally and the fear of contagion in particular. Anticipated stigma is strong in St. Clare with participants expecting people to gossip and thus single out HIV+ members of the community, to avoid sitting next to an HIV+ person, to eschew holding the hand of an HIV+ person during the peace, and to refuse to drink from the challis if they expect that an HIV+ person drank from it. The label “being HIV” leads to ostracism and social isolation.

Many interviewees commented also on the availability of HIV information and education, as well as HIV treatment and support. HIV help and medication are perceived as being accessible and available. Availability leads to the idea that no one has an excuse not to know. With the available education, HIV is seen as a choice. Condoms are felt to be freely available in a number of places. There is, however, the feeling that people do not want to listen to education, that especially young people are not taking the opportunity, and that even though everyone knows about HIV prevention and the importance of using condoms, people refuse to follow the good advice. Interviewees suggest that it is through a lifestyle of clubbing and drugs that people are being infected. In other words, HIV + people have to blame themselves and accept the consequences of their actions. Responsibility is shifted to the infected person, and blame is assigned to those who should have known better.

## Discussion

There was a banner saying “HIV friendly church” in the Anglican Church in Ocean View. In spring 2016 the banner was gone, and no one really knew why it was gone or appeared to have missed it much. The banner was removed when the church interior was redecorated and has simply not come up again. It was not a conscious decision by anyone, rather something that just happened.

The story of the banner is illustrative for the engagement with HIV in the Anglican Church in Ocean View and the wider dioceses of Cape Town and False Bay. When HIV was a “buzz”, when the Anglican AIDS and Healthcare Trust funded local HIV responses, Fikelela founded HIV Task Teams around the diocese, and *Agents of Change* were sent out to local congregations, there was large involvement and HIV was considered to be an important issue. However, when funding dried up local initiatives could no longer be supported and as time passed, other issues became important. HIV was replaced from the top of the agenda until it shifted further and further down. Today, people generally know that HIV exists, but it appears far removed. With gangsterism, substance abuse, and shootings rife in Ocean View, other concerns have taken over. HIV is brought up on World AIDS day and is referred to at times in the service, but far from the centre of attention, even if many people still see HIV as an issue that impacts the life of the congregation and the community. HIV is a topic, but not one that receives a lot of attention. As one interviewee put it, there is “no more buzz” around questions of HIV. To put it differently, HIV is still mentioned, but not often talked about. The basic pattern is repeated in practical HIV-related work. Many congregations responded actively to the epidemic and started HIV Task Teams. The difficulty to address HIV, problems in securing funding, and the immense effort needed to sustain a focussed working group have left many of these initiatives fade out and Task Teams to commit themselves to other issues.

The association of HIV with certain groups or lifestyles entails the possibility of HIV conceptualisations that potentially add to stigma and create a false sense of security. There is a danger of conflating cause and effect, and specific behaviours or circumstances are associated with the virus. The difficulty is to distinguish between correct perceptions of key populations at higher risk and harmful conceptualisations of HIV as pertaining only to specific subgroups. A number of groups are usually identified to be more exposed to HIV; these include men who have sex with men, people who inject drugs, people who sell sex, prisoners, transgender people, women, children, and young people and adolescents (AVERT 2016). Identifying people at higher risk of being exposed to HIV can help to foster a more robust response to the epidemic and inform political prioritisations in mitigating the risk of exposure. However, conceptualising HIV as a virus that befalls specific groups can be problematic if it entails a false sense of security for those not identifying as members of those groups. Once it is accepted that group membership in some of the key populations is voluntary, such as men who have sex with men, people who inject drugs, or people who sell sex, it is easy to blame HIV + people for their infection. The understanding of HIV as a consequence of one’s own irresponsible actions works in the same way. Both put the responsibility and the blame on the HIV + person and add to HIV stigma. The problem is compounded by the perceived relationship between HIV and irresponsible behaviour such as promiscuity, unfaithfulness, and unprotected sex. The focus remains on risky behaviour rather than on people living in risky environments.

Interviewees in Ocean View, Lentegeur, and Constantia see the local Anglican Church to be an important part in people’s lives with church leaders being role models within both the congregation and the wider community. People in these parishes have therefore the possibility to make a tangible contribution towards the prevention of HIV. HIV perspectives could be integrated into the current structures and find a place in sermons, prayers, and special prayers. Awareness can also be raised by having specialised HIV services apart from World AIDS day. HIV and sexuality can be addressed with youth during confirmation classes or in Sunday school, equipping young people to protect themselves from the virus. Also, taking up HIV-related work within the congregation again is a way to increase the awareness around HIV and to keep HIV on the agenda.

Today the local Anglican Church displays a complex picture. The efforts to address HIV and to practically engage the epidemic have largely subsided, and there is “no more buzz”. The efforts have, however, borne fruit with a majority of people being aware of HIV. At the same time, HIV + persons do not publicly self-disclose their status in the local congregations and there is a beginning discourse of blame as responsibility is levied on those who should have known better. This points to stigma being alive and well. There is a real danger that HIV stigma is gaining ground again as efforts to oppose stigma subside.

## Conclusion

Only by continuously engaging issues of HIV and AIDS can the awareness of the epidemic be sustained, and people live in recognition of the fact that HIV affects them personally. Continuous *HIV talk* can normalise the infection, reduce stigma, increase the uptake of VTC and treatment, and work towards the prevention of HIV. To move beyond HIV stigma, the perception of HIV needs to move from being related to risky behaviour to being associated with risky environments. Only when HIV is not seen as a choice or as a consequence of imprudent individual behaviour, can HIV conceptualisations change through the practise of HIV talk and work.
